# Three-dimensional Organization of Layered Apical Cytoskeletal Networks Associated with Mouse Airway Tissue Development

**DOI:** 10.1038/srep43783

**Published:** 2017-03-08

**Authors:** Kazuhiro Tateishi, Tomoki Nishida, Kanako Inoue, Sachiko Tsukita

**Affiliations:** 1 Graduate School of Frontier Biosciences and Medicine, Osaka University, Osaka 5650871, Japan; 2 Japan Textile Products Quality and Technology Center, Kobe 6500011, Japan; 3 Research Center for Ultra-high Voltage Electron Microscopy, Osaka University, Osaka 5670047, Japan

## Abstract

The cytoskeleton is an essential cellular component that enables various sophisticated functions of epithelial cells by forming specialized subcellular compartments. However, the functional and structural roles of cytoskeletons in subcellular compartmentalization are still not fully understood. Here we identified a novel network structure consisting of actin filaments, intermediate filaments, and microtubules directly beneath the apical membrane in mouse airway multiciliated cells and in cultured epithelial cells. Three-dimensional imaging by ultra-high voltage electron microscopy and immunofluorescence revealed that the morphological features of each network depended on the cell type and were spatiotemporally integrated in association with tissue development. Detailed analyses using Odf2 mutant mice, which lack ciliary basal feet and apical microtubules, suggested a novel contribution of the intermediate filaments to coordinated ciliary beating. These findings provide a new perspective for viewing epithelial cell differentiation and tissue morphogenesis through the structure and function of apical cytoskeletal networks.

During the development of multicellular organisms, epithelial cells adhere to each other, followed by the formation of various types of cellular asymmetry such as apico-basal polarity and planar cell polarity (PCP)^1^,^2^,^3^. During epithelial cell polarization, various types of cytoskeleton, including actin filaments, intermediate filaments, and microtubules, play specific roles. In addition, the formation of subcellular compartments, highly specialized cellular regions which are indispensable for development^4^, coincide with typical structures containing multiple cytoskeletons, such as the midbody in dividing cells and the leading edge in migrating cells^5^,^6^. Although these cytoskeletal “superstructures” are involved in sophisticated cellular functions, high-resolution and three-dimensional information revealing their organization, including their changes during tissue maturation, is still fragmentary.

Multiciliated cells (MCCs) in the airway bear hundreds of cilia, which project from basal bodies (BBs) on their apical surface, and generate mucus flow on the tracheal surface by beating these cilia in a coordinated and unidirectional manner. Mucociliary transport, which occurs after the maturation of MCCs and whole tracheal tissue, functions as a primary protection system against various infections. Since their structure and material were revealed, the MCCs of various species and/or tissues have been used to explore the roles of cytoskeletons in subcellular compartmentalization and cell differentiation.

Actin filaments and actin interacting proteins, and their dynamic reorganizations driven by cellular signalling are implicated in BB migration and docking during ciliogenesis^7^. Microtubules in the apical region of mouse tracheal MCCs control the relative position and orientation of each BB via the basal foot (BF), which is conical structure associated with the BBs. Microtubule polarization occurs early in Drosophila development, in parallel with the asymmetric accumulation of PCP proteins^8^.

Although many studies have focused on the function of microtubules in MCCs^9^,^10^,^11^,^12^, little is known about their three-dimensional organization or their interrelationship with other types of filaments. Moreover, there is little information about general roles of the cytoskeletal superstructures that form the apical subcellular compartment in epithelial cells. In the present study, we defined the apical cytoskeletal networks that comprise the complex cytoskeletal superstructure beneath the apical membrane. Three-dimensional examinations revealed actin, intermediate filament, and microtubule networks, in the apical compartment that is provided by the continuous strand of tight junctions^13^,^14^. Ultra-high voltage electron microscopy and super-resolution confocal microscopy revealed distinct differences in the three-dimensional location and spatiotemporal regulation of each type of filament in the apical cytoskeletal networks of developing tracheal cells. In addition, similar network structures were found in nonciliated tracheal cells and cultured epithelial cells. Our results reveal that apical cytoskeletal networks, one of the cytoskeletal superstructures may generally contribute to the formation of specific apical compartments in epithelial cells and epithelial development.

## Results and Discussion

### Layered network structure in airway epithelial cells

**Figure 1:**
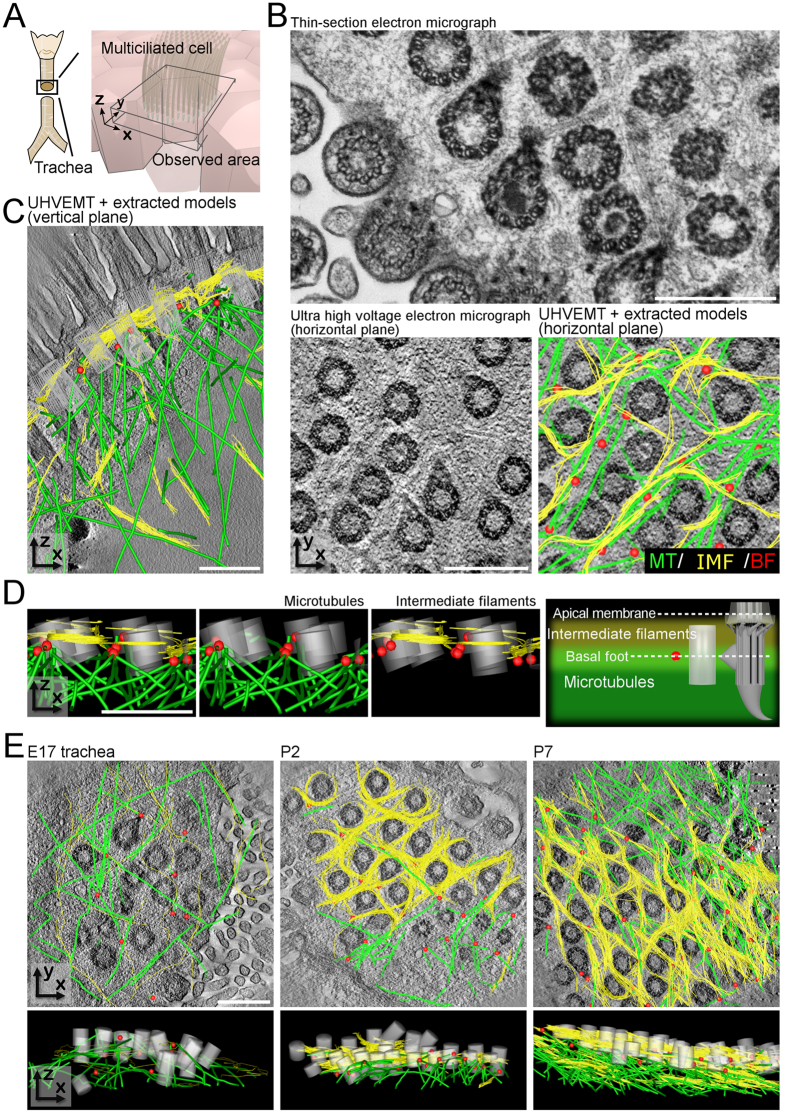
Apical cytoskeletal networks directly under the surface of mouse tracheal MCCs. (**A**) Schematic of the mouse tracheal surface. (**B**) Horizontal plane TEM and UHVEMT images of adult mouse tracheal MCCs. Conventional TEM image (top), reconstituted UHVEMT image (bottom left) and reconstituted UHVEMT image with extracted models (bottom right). See also Figure S1A. MT: Microtubule, IMF: Intermediate filament, BF: Basal foot. (**C)** Reconstituted vertical plane UHVEMT image of an adult mouse tracheal MCC with extracted models. See also Figure S1B and Movie 1. (**D**) Z-profile extracted models of BBs, BFs, and the apical cytoskeletal networks of microtubules and intermediate filament networks (left three). Schematic of the position of each cytoskeleton (right). (**E**) Reconstituted horizontal plane UHVEMT images with extracted models (top) and z-profile extracted models (bottom) of developing mouse tracheal MCCs. See also Movie 2. Microtubules, intermediate filaments, BBs, and the tips of BFs are pseudocoloured green, yellow, grey, and red, respectively. Scale bars represent 500 nm.

The apical regions of MCCs are exposed to mechanical stress because they bear hundreds of synchronously beating cilia (Fig. 1A)^15^,^16^. Early EM studies showed dense cytoskeletons surrounding the BBs in various ciliated cells^17^,^18^,^19^. However, the three-dimensional cytoskeletal structure was unclear. To examine the spatial arrangement of the cytoskeletons in MCCs, we first carefully observed mature mouse trachea tissues with ultra-high voltage electron microscopic tomography (UHVEMT). Due to its extremely high power capacity, UHVEMT enables us to analyse thick samples three-dimensionally with high resolution (see Materials and Methods for more information). While the presence of dense microtubule structures surrounding ciliary BBs was previously reported^20^, UHVEMT revealed that intermediate filaments also formed a network structure between each BB, and that the intermediate filaments and microtubules were structurally associated with each other (Figs 1B and S1A).

Although both filaments formed a network structure along the horizontal plane in the apical region of MCCs, the microtubule and intermediate filament networks showed three major differences (Figs 1C,D and S1B, Movie S1). First, the intermediate filament network was positioned just under the membrane to the BF level (50–200 nm deep from the apical surface), while the microtubule network was deeper, beneath the BF level (more than 200 nm deep). Second, the intermediate filaments formed a thin network that was almost two-dimensional, while the microtubules formed a thick, complex, three-dimensional network. Finally, owing to differences in their material properties^21^,^22^, the intermediate filament network formed a circular, round mesh closely around each BB, while the microtubule network had a polygonal mesh (Fig. S1C).

To investigate the formation process of the apical cytoskeletal networks, we examined the developing mouse trachea using UHVEMT. The three-dimensional organizations of microtubules and intermediate filaments changed together as the tissue matured (Fig. 1E and Movie S2). In CACO-2 colonic adenocarcinoma cells, intermediate filaments accumulate at the apical membrane prior to brush border formation^23^. In tracheal MCCs, we observed that the microtubules formed a sparse network around embryonic day 17 (E17) (migrating BBs begin to dock to the apical membrane at this stage), while only a few intermediate filaments were detected at this stage. Continuous, but relatively sparse networks of intermediate filaments were observed from postnatal day 2 (P2), while the microtubules formed an immature network. The filaments steadily increased, until both filament networks appeared to reach a steady state, forming dense meshes around each BB with little further change, around P7-P9. Although UHVEMT enabled us to reveal the changes in intermediate filament and microtubule networks during tracheal differentiation, the details of these transitions await future studies, because of the limitation of the temporal resolution in UHVEMT.

### Formation and polarization of apical cytoskeletal networks accompanying tracheal development

**Figure 2:**
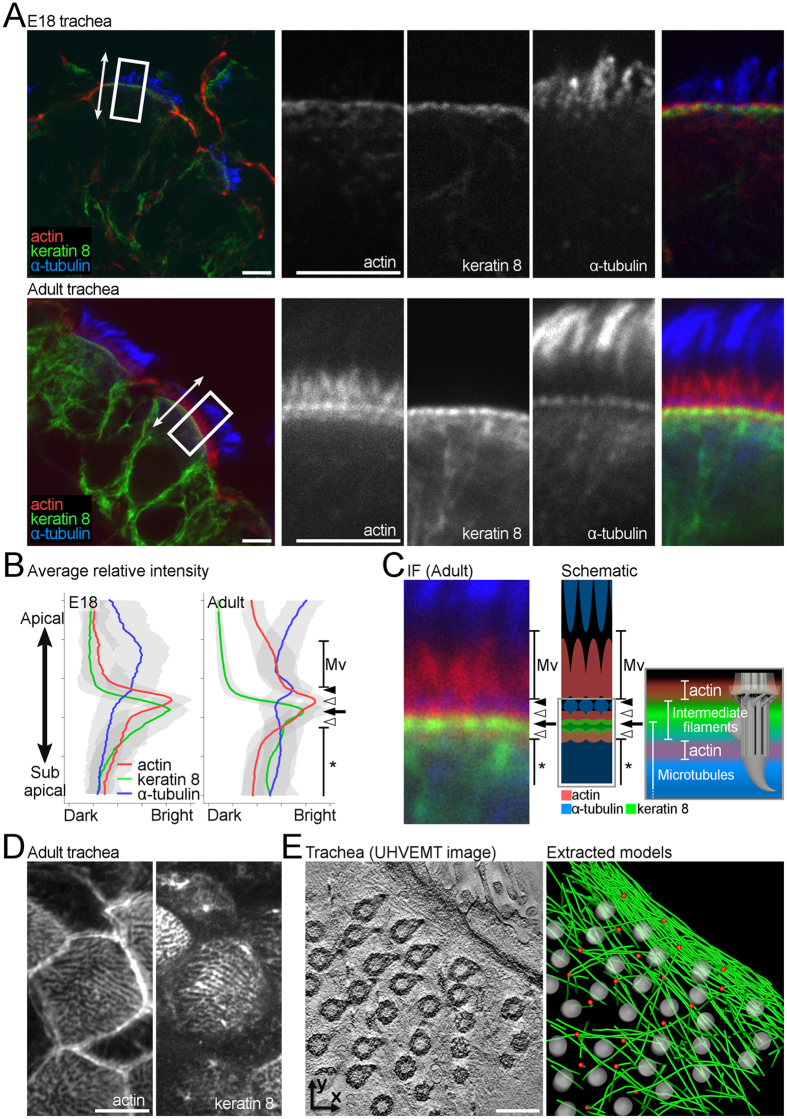
Spatial pattern and temporal changes in the apical cytoskeletal networks in developing mouse trachea. (**A**) Immunofluorescence of embryonic day 18 and adult mouse tracheal MCCs in the vertical plane. The white arrows and rectangles indicate the apico-basal axis and magnified regions in right panels respectively. See also Figure S2A. (**B**) Average relative fluorescence intensity of different cytoskeletons in MCCs at different developmental stages. Coloured lines and grey bands represent the average relative intensity and SEM, respectively. At least 300 linear ROIs were analysed in more than 5 cells. Black arrowhead, white arrowhead, and black arrow indicate the peaks of α-tubulin, actin, and keratin 8, respectively. Asterisk indicates the microtubule network. See also Figure S2B–D. (**C**) Immunofluorescence (IF; left) and schematic (right) of the apical cytoskeletal networks. Black arrowhead, white arrowhead, and black arrow indicate the peaks of α-tubulin, actin, and keratin 8, respectively. Asterisk indicates the microtubule network. See also Figure S2B–D. **(D**) Immunofluorescence of adult mouse tracheal MCCs in the horizontal plane. See also Figure S4A. (**E**) Reconstituted horizontal plane UHVEMT image of tracheal MCC (left) and extracted model of the polarized microtubule network (right). Microtubules, BBs, and the tips of BFs are pseudocoloured green, grey, and red, respectively. See also Movie 3. Scale bars represent 5 μm (**A**,**D**) and 500 nm (**E**). Mv: microvilli.

Actin filaments are known to be important for MCC maturation^7^,^10^. To better understand the roles of the apical cytoskeletal networks as the cytoskeletal superstructure, we next analysed the spatiotemporal establishment of actin filaments, in addition to intermediate filaments and microtubules in the developing mouse trachea. Changes in the relative positions of actin, keratin 8, and α-tubulin immunofluorescence signals in the apical region were observed from E18 to adulthood (20 weeks). In Xenopus epidermis, two pools of cortical actin filaments are associated with ciliary BBs via the typical adhesion complex, and function to elicit proper BB spacing^9^,^10^. In the adult mouse tracheal MCCs, however, we observed three intense signals: two layers in the apical region and a microvillus-derived signal on the surface (Figs 2A,B and S2). We previously reported that the effects of depolymerizing reagents in cultured mouse MCCs are different from those in Xenopus^24^. Several differences between the Xenopus epidermis and mouse tracheal MCCs, such as the ciliary density and the presence of microvilli, could explain these variations. The structural and functional comparison of the actin filament layers observed in our analyses and the pools of cortical actin filaments in Xenopus remains a topic for future study.

Keratin 8, one of the major keratins in simple epithelia, showed an intense signal at the apical region and a weak signal in a deeper region. Most simple epithelia, such as those in the intestine, kidney, and uterus, exhibit a dense intermediate filament layer directly beneath the apical membrane, with little filament formation in the cytoplasmic region^25^,^26^,^27^. In mouse tracheal MCCs, however, a relatively weak filamentous cytoplasmic signal was also observed in addition to the strong signal at the apical membrane, as reported in some other epithelial tissues^28^,^29^. Consistent with our UHVEMT observations, intermediate filaments started to form networks around P2–4. Simultaneous with the network formation, an increase in number of ciliated cells in the trachea was detected, followed by the establishment of mucociliary transport (Fig. S3).

Staining for α-tubulin showed an intense signal in the apical region and a broad signal in a deeper region in addition to the strong, cilia-derived signal. Our UHVEMT analyses revealed two types of microtubules in mature MCCs: those extending between BBs in the horizontal plane, and those extending vertically toward the basal region. The vertical microtubules began to increase at a later stage (P7–P9), in contrast to the gradual enrichment of the horizontal microtubules. Consistent with these ultrastructural observations, the broad signals beneath the apical membrane were detected by immunofluorescence only in the late stages of multiciliogenesis. We previously reported that microtubules direct the proper alignment of BBs and that the velocity of each BB slows at a certain stage of MCC maturation^24^. These horizontal and vertical microtubules may function as a fence for BB pattern formation and as an anchor for the rigid ciliary base, respectively.

To further clarify the roles of these networks, we analysed the establishment of each filament type by immunofluorescent staining in the horizontal plane. Owing to the presence of cilia, spatial information about the microtubules in the apical region of MCCs with immunofluorescence was limited. On the other hand, actin and intermediate filament network structures surrounding the BBs were observed (Fig. S4A). Collectively, our findings showed that the actin, intermediate filaments, and microtubules formed layered networks around the ciliary BBs (Figs 2C,D and S4B), and that the networks saturated around P7. The speed and directionality of cilia-generated flow in the mouse trachea are rapidly established during P6–9^30^. Our structural and functional analyses suggested that the establishment of cytoskeletal superstructures promotes the acquisition of tissue-level functions, such as mucociliary transport in the airway.

The polarization of microtubules along the horizontal plane has been reported in the epithelial cells of various organisms^8^,^11^,^12^,^31^. Our UHVEMT analyses revealed the detailed structure of the polarized microtubules in MCCs (Fig. 2E and Movie S3). This microtubule accumulation, which is thought to occur after the establishment of PCP, was found on the side of mature MCCs toward which the BFs point, and the most microtubules were distributed parallel to the cell adhesion. Some studies suggest that mechanical strain is a controlling factor for both the acquisition and tuning of the planar polarization in MCCs^16^,^31^. In these cells, ciliary movements and cilia-generated external fluid flow are possible regulators of polarization in addition to PCP-derived cues. We recently reported that the microtubule-associated protein cingulin, which is known as a component of tight junctions^32^ anchors microtubules to tight junctions parallel to each other in the apical plane^33^. In the trachea, cingulin showed this localization in both early-stage (E18) and mature MCCs (Fig. S5A). In addition, a lateral association of intermediate filaments with cell junctions was observed by UHVEMT (Fig. S5B). These results suggest that additional components such as cingulin organize cytoskeletal networks including polarized microtubules at the junctional complex, and facilitate the apico-basal and planar cell polarizations.

### Formation of intermediate filament networks even in the absence of BF-associated microtubules

**Figure 3:**
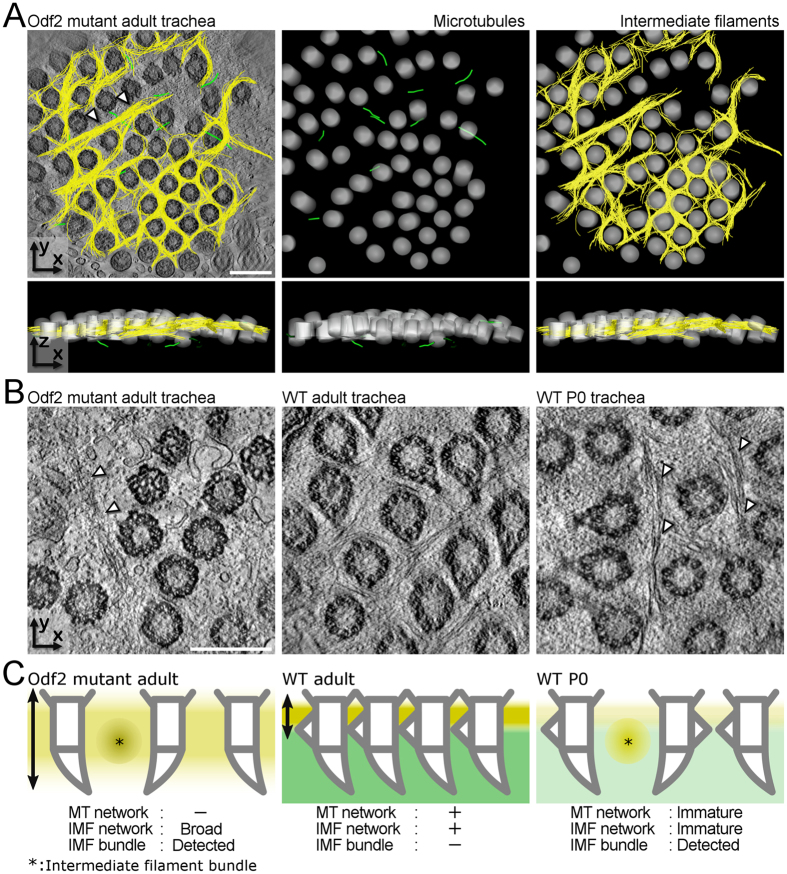
Apical intermediate filament network in the absence of apical microtubules in the Odf2 mutant mouse. (**A**) Extracted models of microtubules and intermediate filament networks (top) and z-profile extracted models (bottom) of the Odf2 mutant mouse tracheal MCCs. Arrowheads indicate a intermediate filament bundle. Microtubules, intermediate filaments and BBs are pseudocoloured green, yellow and grey respectively. See also Movie 4. (**B)** Reconstituted UHVEMT images of the horizontal plane of Odf2 mutant adult, wild-type adult, and neonatal mouse tracheal MCCs. Arrowheads indicate intermediate filament bundles. (**C**) Schematic of intermediate filament and microtubule networks in the Odf2 mutant adult, wild-type adult, and neonatal tracheal MCCs. Green and yellow indicate microtubule and intermediate filament networks respectively. Asterisks indicate intermediate filament bundles. Scale bars represent 500 nm.

To clarify the relationship between the microtubules and intermediate filaments in the apical cytoskeletal networks, we used Odf2 mutant mice which lack exons 6 and 7 in Odf2 gene^20^. Odf2 is a major component of the mother centriole and ciliary BBs^34^,^35^. In these mice, the transitional fibres that organize membrane fusion during ciliogenesis, are formed while the ciliary BFs and BF-associated microtubules are absent^20^. Our UHVEMT analysis of Odf2 mutant MCCs revealed that in the absence of the apical microtubule network, the intermediate filaments formed network structures around the BBs (Figs 3A and S5C). We previously reported that Odf2 mutant cultured cells lacking centriole subdistal appendages which are homologous to ciliary basal feet, have a low tolerance for depolymerizing stress by the treatments^36^. Together, these results strongly suggest that ciliary basal feet contribute to the formation and/or retention of microtubule networks. In the apical regions of MCCs, the BBs are exposed to mechanical stress caused by ciliary beating. It is generally accepted that mechanical stress promotes the deploymerization of microtubules^37^,^38^, and the enrichment of intermediate filament networks^39^,^40^,^41^. Therefore, the scant microtubule and thick intermediate filament networks around the BBs in BF-lacking Odf2 mutant mice are thought to result from mechanical stress.

In wild-type mouse MCCs, bundles (unbranched thick accumulations) of intermediate filaments were only seen in the early stages (by P2), before the establishment of the dense network. In Odf2 mutant MCCs, however, the intermediate filaments formed bundles between BB clusters, even in the adult. Due to the physical nature of intermediate filaments, the larger gaps between BB clusters in Odf2 mutant MCCs enabled the bundle formation (Fig. 3B)^42^. Moreover, the intermediate filament network was distributed more broadly in the vertical direction in the Odf2 mutant MCCs, although the horizontal image was similar to that of mature MCCs in the wild-type tissue (Fig. 3C and Movie S4). Collectively, these two morphological events, the change of the intermediate filament network and the bundle formation in wider BB gaps, may compensate for the loss of the microtubule network around BBs^43^, to support ciliary beating and mucociliary transport in the trachea of Odf2 mutant mice.

The roles of intermediate filaments in MCCs are largely unknown. Our UHVEMT-based observations in the developing trachea showed that intermediate filament networks formed prior to the microtubule networks. In the Odf2 mutant, intermediate filament networks formed in the apical microtubule-lacking MCCs. These two observations suggest that the microtubule-network formation is associated with the pre-existing intermediate filaments. In addition, the existence of cytoskeletal linker proteins such as plectin in the apical cytoskeletal networks supports the idea that mutual regulation between intermediate filaments, actin filaments, and microtubules plays a critical role in their function and organization, along with the specific functions of each filament network^24^. A deeper understanding of the mechanisms by which intermediate filaments control other filaments and/or the differentiation of tracheal MCCs should provide novel insight into the functions of these filaments. Detailed investigations addressing these questions are underway.

### Apical cytoskeletal networks in other epithelial cells

**Figure 4:**
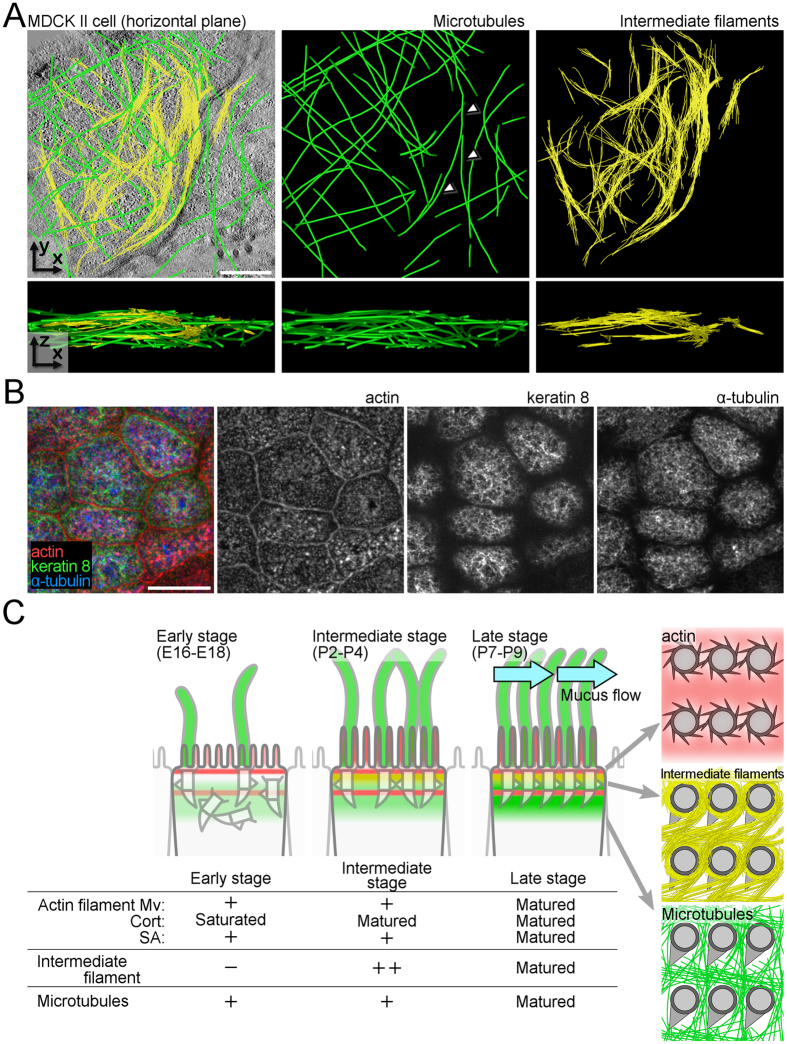
Apical cytoskeletal networks in cultured epithelial cells. (**A**) Extracted models of microtubules and intermediate filament networks (top) and z-profile extracted models (bottom) of cultured MDCK cell. Microtubules, intermediate filaments and BBs are pseudocoloured green, yellow and grey respectively. Arrowheads indicate microtubules that extend in parallel with cell junctions. See also Movie 5. (**B**) Immunofluorescence of cultured MDCK cells. (**C)** Schematic of the apical cytoskeletal network formation in mouse tracheal MCCs. Stepwise transition in the longitudinal axis is shown in left panels and table, and the stereotypic network patterns are shown in the right panels. Mv: microvilli, Cort: cortical actin, SA: subapical actin. Scale bars represent 500 nm (**A**) and 5 μm (**B**).

To explore the general function of apical cytoskeletal networks, we next examined Madin-Darby canine kidney (MDCK) cells, a well-characterized cultured epithelial cell line, as a model system of epithelial tissue. Although the layered distribution was less obvious compared to tracheal MCCs, UHVEMT analysis revealed networks consisting of intermediate filaments and microtubules in the apical region (Fig. 4A and Movie S5). Consistent with our previous study^33^, side-by-side association of microtubules with cell junctions were detected. The intermediate filament networks formed circular meshes with a larger diameter than those observed in MCCs. These findings suggested that a common cellular cue exists in tracheal MCCs and cultured cells that drives the circular pattern formation of intermediate filaments. The dynamics and stiffness of intermediate filaments can change dramatically by conditions such as their crosslinking or ability to slide across each other^21^. Bundled intermediate filaments are frequently observed in cultured cells and may contribute to the difference of the radius of mesh by stiffening each filament. Although the appearance of each mesh was relatively unclear, immunofluorescence also revealed network-like patterns consisting of actin, intermediate filaments, and microtubules in the cultured cells (Fig. 4B). Furthermore, the nonciliated cells of the mouse trachea showed network-like structures consisting of actin, intermediate filaments, and microtubules by immunofluorescence (Fig. S5D). It is generally accepted that actin-based membrane-associated cytoskeletons are responsible for the distributions of some transporters and channels in the apical membrane^44^. In intestinal epithelial cells, an essential role of intermediate filaments in the proper distribution of ion transporters on the apical surface was reported^45^. In addition, the presence and possible functions of an apical sub-membrane keratin filament network in several cultured epithelial systems were addressed^46^,^47^. These results indicate that apical cytoskeletal networks, which are highly developed in tracheal MCCs, may be ubiquitous structures in epithelial tissues.

## Conclusions

In the present study, we identified the three-dimensional organization of the apical cytoskeletal networks and their developmental processes that accompany the tissue maturation of mouse tracheal MCCs (Fig. 4C). These networks formed layers with actin on the apical surface, followed by intermediate filaments, and microtubules, and showed distinct spatiotemporal transitions during development. The functional maturation of MCCs coincided with the establishment of the apical cytoskeletal networks, suggesting that this superstructure is important for generating the apical compartments involved in developmental processes. The concurrent establishment of the apical cytoskeletal networks and function of MCCs indicates the value of spatiotemporal analyses that reveal the pattern transition of each cytoskeletal filament, as well as its expression level. Our observation of the apical cytoskeletal networks in other epithelial cells suggests the existence of a general mechanism in which these networks contribute not only to form, but also the maintenance of tissue-specific functions by promoting apical compartmentalization in cooperation with tight junctions. The association of apical cytoskeletal networks with epithelial cell compartmentalization highlights that high-resolution structural examinations of cytoskeletal superstructures will yield crucial information for various studies of cellular differentiation and tissue development.

## Methods

### Electron microscopy

All samples were prepared based on a classical method^48^. After isolating the trachea or cultured cells, the samples were fixed with 0.1% tannic acid, 2% formaldehyde, and 2.5% glutaraldehyde in 100 mM Hepes buffer (pH 7.5) for 1 h at 37 °C, followed by post fixation with 1% OsO_4_ in 100 mM Hepes buffer (pH 7.5) for 2 h on ice. The samples were dehydrated and embedded in Poly/Bed 812 (Polysciences). For transmission electron microscopy, the samples were serially sectioned at 50 or 70 nm and analysed by a JEM-1400 plus (JEOL). For ultra-high voltage electron microscopic tomography (UHVEMT), the samples were sectioned at 700 nm and mounted on formvar-coated 50-mesh copper grids (NISSHIN-EM). Colloidal gold particles (20-nm diameter) were deposited on both sides of each section, and the samples were observed at 1 MeV acceleration voltage (H-3000; Hitachi). Images were taken at 25 K×, from −60 to +60 degrees at 2-degree intervals around a single tilt axis, and acquired with an SSCCD camera (model F415S; TVIPS GmbH). Image calibration and 3D reconstructions of each series were performed using IMOD software^49^.

### Antibodies

Rat anti-Odf2 mAb, rabbit anti-Odf2 pAb, rat anti-centriolin mAb, rat anti-cingulin mAb, and mouse anti-ZO-1 mAb were generated previously. Mouse anti-α-tubulin mAb (Sigma) was purchased. Rat anti-keratin 8 mAb was generated from the culture supernatant of TROMA-I hybridoma (DSHB, University of Iowa). All primary antibodies were used at 100× −1000× dilution. Alexa Fluor 488–, 568–, and 647–labelled secondary antibodies were purchased (Invitrogen). All secondary antibodies were used at a dilution of 1,000×.

### Immunofluorescence

The cells or isolated tissues were fixed with 37 °C 4% paraformaldehyde in PBS or with ice-cold methanol for 10 min, washed with PBS three times, and permeabilized with 0.5% Triton X-100 in PBS for 15 min. The samples were then washed with PBS, soaked in 1% BSA in PBS for 30 min at RT, and incubated with primary antibodies for 1 h at 37 °C. The samples were then washed with PBS, incubated with Alexa Fluor 488-, 555-, or 647- labelled secondary antibodies (Invitrogen) for 1 h at 37 °C, washed with MQ, and mounted in fluorescence mounting medium (Dako). Images were obtained with a Spinning Disk-Olympus Super Resolution microscope (SD-OSR, Olympus)^50^ with a UPlan SApo 60x NA1.3 silicon oil-immersion objective and ORCA-Flash 4.0 v2 sCMOS (Hamamatsu). Images were prepared using Adobe Photoshop, Illustrator (Adobe Systems), and ImageJ (National Institutes of Health).

### Animal experiments

C57BL/6J (B6J) mice (Japan SLC) were used for all experiments. Animal experiments were performed according to the declaration of Helsinki and protocols approved by the Osaka University.

### Cell culture

Madin-Darby canine kidney (MDCK) cells were used for the experiments with cultured cells. Cells were seeded onto 12-well gelatine-coated Transwell plates (Corning) at high density (1 × 10^5^ cells/well) and grown in Dulbecco’s modified Eagle medium supplemented with 10% FBS. For both electron microscopic and immunofluorescence studies, the cells were cultured for 5 days before each experiment to establish apico-basal polarity.

### Quantification of relative fluorescence intensity in MCCs

All analyses were performed with ImageJ (National Institutes of Health) (for the image analysis), SciLab (Scilab Enterprises), and R (The R Foundation) (for the statistical analyses and plotting). At least 300 linear ROIs were analysed in more than 5 cells for each developmental stage. See Supplemental Experimental Procedures for the detail information.

## Additional Information

**How to cite this article:** Tateishi, K. *et al*. Three-dimensional Organization of Layered Apical Cytoskeletal Networks Associated with Mouse Airway Tissue Development. *Sci. Rep.* **7**, 43783; doi: 10.1038/srep43783 (2017).

**Publisher's note:** Springer Nature remains neutral with regard to jurisdictional claims in published maps and institutional affiliations.

## Supplementary Material

Supplementary Information

Supplementary Movie 1

Supplementary Movie 2

Supplementary Movie 3

Supplementary Movie 4

Supplementary Movie 5
