# Supplementary material for: Three-dimensional Organization of Layered Apical Cytoskeletal Networks Associated with Mouse Airway Tissue Development
Source: Sci Rep. 2017 Mar 8;7:43783. doi: 10.1038/srep43783 (PMC5363704; doi:10.1038/srep43783)
Supplement: Supplementary Information [file srep43783-s1.pdf]

Three-dimensional Organization of Layered Apical Cytoskeletal Networks Associated with Mouse Airway Tissue Development

Kazuhiro Tateishi[1], Tomoki Nishida[1,2], Kanako Inoue[3], and Sachiko Tsukita[1]*
[1] Graduate School of Frontier Biosciences and Medicine, Osaka University, Osaka 5650871, Japan
[2] Japan Textile Products Quality and Technology Center, Kobe 6500011, Japan
[3] Research Center for Ultra-high Voltage Electron Microscopy, Osaka University, Osaka 5670047, Japan

*Correspondence to Sachiko Tsukita: atsukita@biosci.med.osaka-u.ac.jp

Supplemental Information

Supplemental Experimental Procedures

Quantification of relative fluorescence intensity in MCCs

All analyses were performed with ImageJ (National Institutes of Health) (for the image analysis), SciLab (Scilab Enterprises), and R (The R Foundation) (for the statistical analyses and plotting).

At least 300 linear ROIs were analysed in more than 5 cells for each developmental stage. The length of each ROI was set as 4 μm, perpendicular to the cell surface. The midpoint was set at the cortical actin. After the measurement of fluorescence intensity in each ROI, the normalisation and the average relative intensity calculation were performed with a custom program written in R.

Functional analyses of MCCs

For the analyses of cell area, number of cilia/cell, and MCCs/total cells, the fluorescent signals of ZO-1, centriolin, and Odf2 were used as indicators of the cell boundary, ciliary basal bodies, and ciliated cells, respectively. Images were acquired with an Orca-ER CCD camera (Hamamatsu) with a BX51 (Olympus) 40× objective. The cell area of each image was calculated after banalisation, which was based on watershed segmentation. The cilia were counted with local maxima selection. For the cell area and MCC ratio analyses, at least 1,000 cells from three different experiments in each developmental stage were analysed. For the cilium number analysis, at least 100 MCCs from three different experiments in each developmental stage were analysed.

The ciliary beat frequency (CBF) and mucociliary transport (MCT) analyses were based on a previous study[1]. For the CBF analysis, images of mouse tracheal MCCs were

acquired with a high-speed camera (FASTCAM MC2.1; Photron) with a Zeiss Axioplan (Carl Zeiss) 63× objective. The CBF was analysed with FFT-based custom programs written in ImageJ and SciLab. For the MCT analysis, fluorescent beads (Fluoresbrite, 0.5 μm; Polysciences) were placed on the tissue surface and recorded with an Orca-ER CCD camera (Hamamatsu) with a BX51 (Olympus) 20× objective. Each video was analysed with Particle Tracker 2D/3D (ImageJ plugin) to get the mucus flow velocity. For the CBF and the MCT analyses, at least 100 MCCs from three different experiments in each developmental stage were analysed.

Supplemental Figures

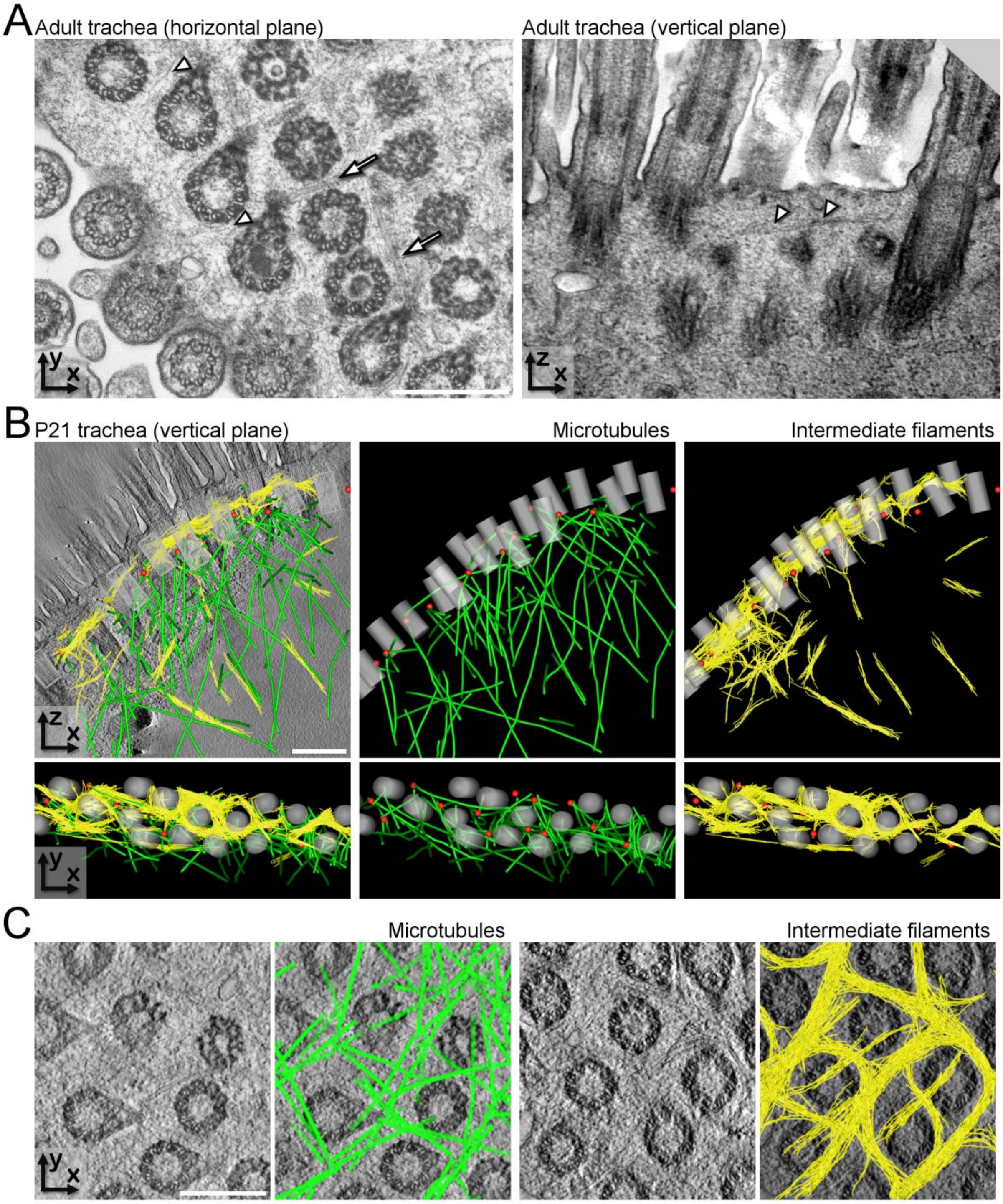

Figure S1 Tateishi et al

Figure S1

Distribution and structure of apical cytoskeletal networks

(related to Figure 1)

A Horizontal and vertical thin-section TEM images of the apical cytoskeletal network in tracheal MCCs.

Arrows and arrowheads indicate microtubules and intermediate filaments, respectively.

B Reconstituted UHVEMT images and extracted models of adult mouse tracheal MCCs.

See also Movie 1.

C Horizontal UHVEMT images and extracted models of microtubules (left) and intermediate filaments (right).

Microtubules, intermediate filaments, BBs, and tips of BFs are pseudocoloured green, yellow, grey, and red, respectively.

Scale bars represent 500 nm.

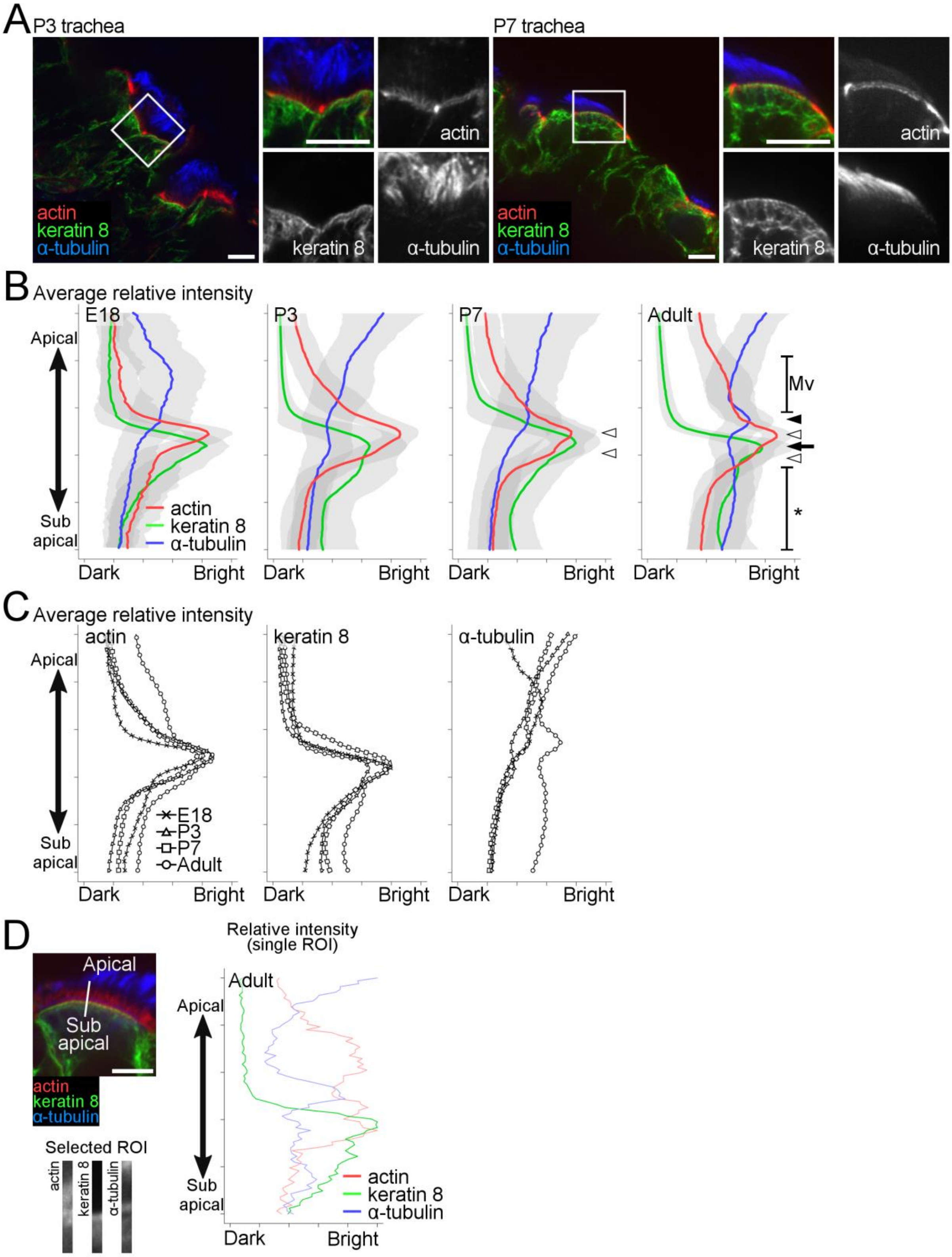

Figure S2 Tateishi et al

Figure S2

Quantitative analysis of developing mouse tracheal MCCs

(related to Figure 2)

A Immunofluorescence of developing mouse tracheal MCCs in the vertical plane. The white rectangles indicate magnified regions in right panels.

B Average relative fluorescent intensity of different cytoskeletons in MCCs at different developmental stages. Coloured lines and grey bands represent the average relative intensity and SEM, respectively. At least 100 linear ROIs from 10 cells were analysed. Black arrowhead, white arrowhead, and black arrow indicate the peaks of α-tubulin, actin, and keratin 8 respectively. Asterisk indicates the apical microtubule network.

C Average relative fluorescent intensity of each cytoskeleton in MCCs at different developmental stages. Lines with xs, triangles, squares and circles indicate E18, P3, P7 and Adult respectively.

D Quantified fluorescent intensity of the different cytoskeletons in MCCs. Example of an analysed image (top left) and each channel of the selected ROI (bottom left). Relative intensity of actin, intermediate filament, and microtubules in the selected ROI is shown by red, green, and blue lines, respectively (right). See also Supplemental Experimental Procedures.

Scale bars represent 5 μm.

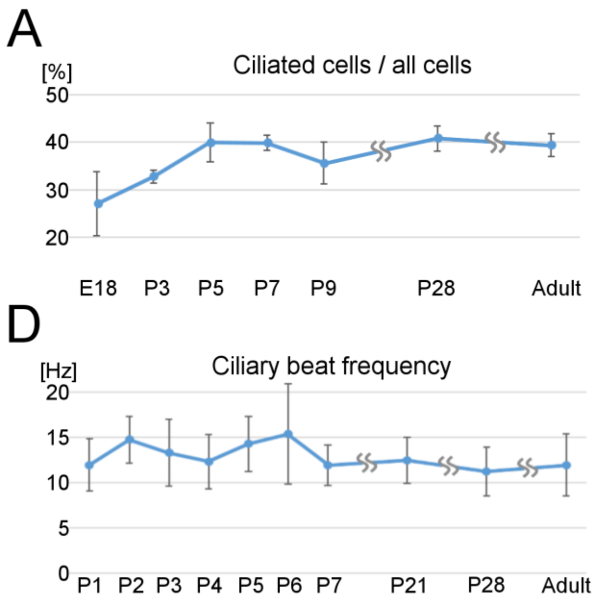

Figure S3 Tateishi et al

Figure S3

Functional analyses of MCC maturation.

(related to Figures 2)

A Ratio of ciliated cells in all cells in each developmental stage.

B Number of cilium of MCCs in each developmental stage.

C Apical surface area of MCCs in each developmental stage.

D Ciliary beat frequency in each developmental stage.

E Mucus flow velocity of tracheal surface in each developmental stage.

Error bars: SD, N.D.: not detected

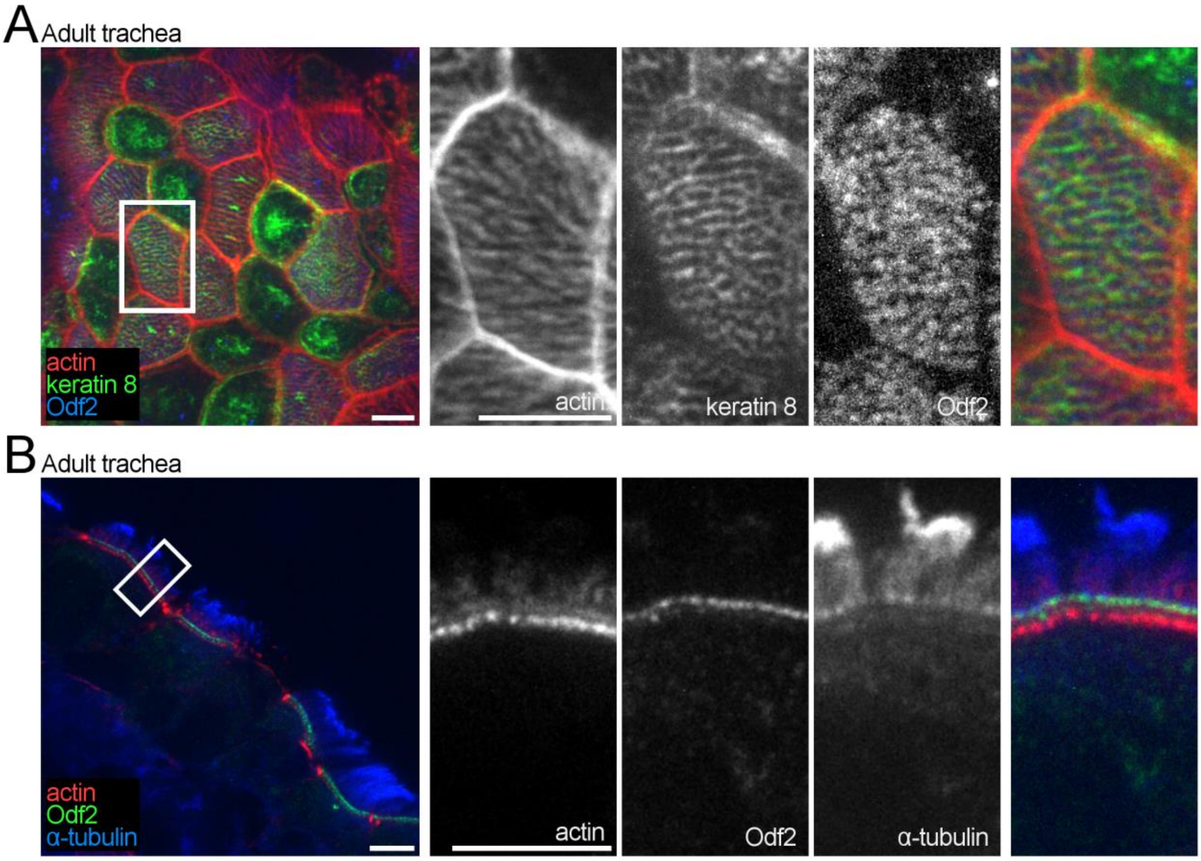

Figure S4 Tateishi et al

Figure S4

Immunofluorescence of mouse tracheal cells

(related to Figures 2)

A Immunofluorescence of adult mouse trachea in the horizontal plane.

B Immunofluorescence of adult mouse trachea in the vertical plane.

Scale bars represent 5 μm.

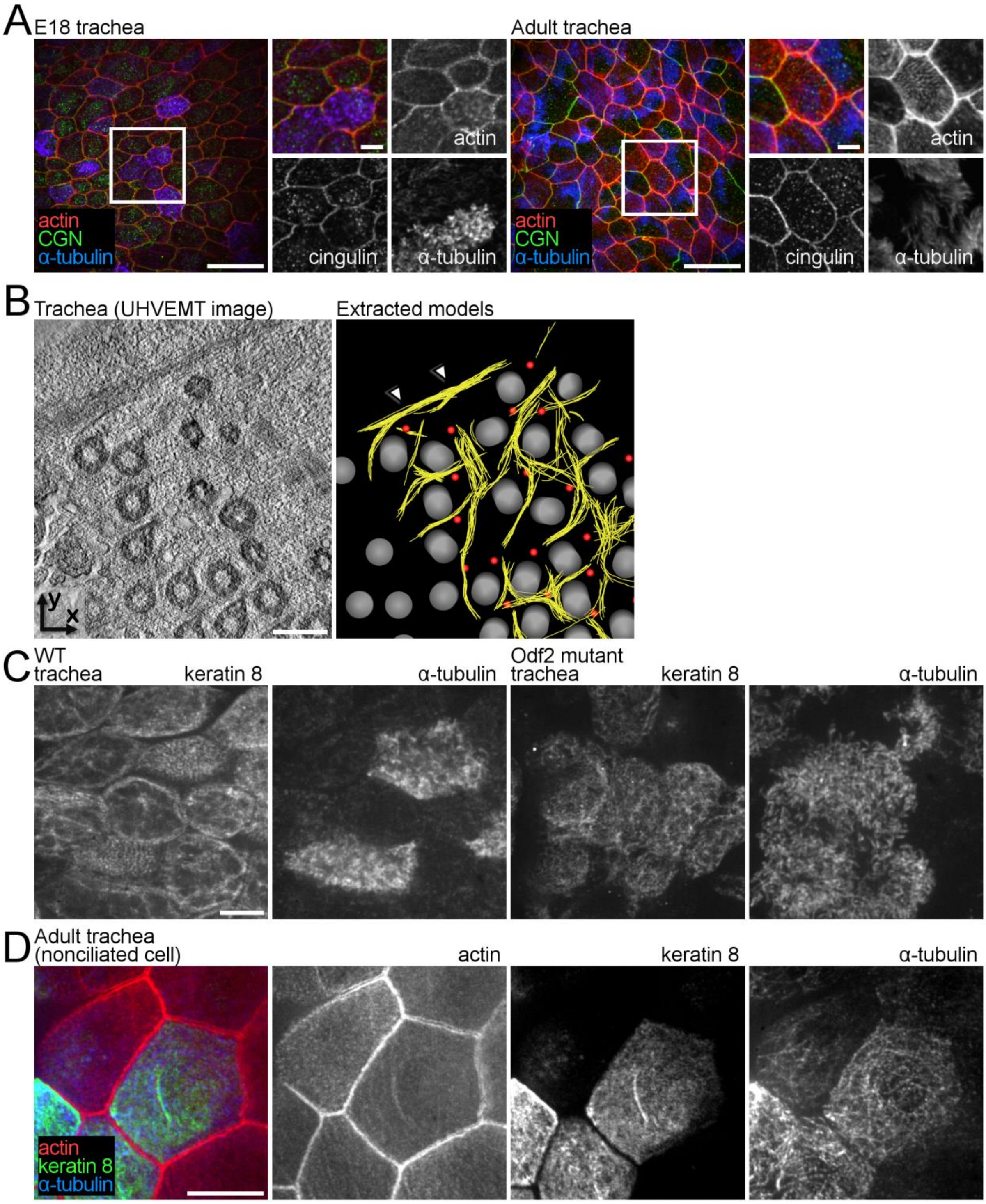

Figure S5 Tateishi et al

Figure S5

Immunofluorescence and UHVEMT images of mouse tracheal cells

(related to Figures 2 and 4)

A Immunofluorescence of mouse tracheal MCCs on embryonic day 18 (top) and in adult (bottom) in the horizontal plane.

B Reconstituted horizontal plane UHVEMT image of tracheal MCC (left) and extracted model of intermediate filament network (right). intermediate filaments, BBs, and the tips of BFs are pseudocoloured yellow, grey, and red, respectively. White arrowheads indicate the cell-cell region of observed cells.

C Immunofluorescence of wild-type and Odf2 mutant mouse tracheal MCCs in the horizontal plane.

D Immunofluorescence of adult mouse tracheal nonciliated cells in the horizontal plane.

Scale bars represent 5 μm (A, C and D), 500 nm (B).

Supplemental Movies

1. Reconstituted ultra-high voltage electron microscopic tomography images with extracted three-dimensional models of the apical cytoskeletal networks and ciliary basal bodies in postnatal day 21 mouse tracheal MCCs in the vertical plane. Microtubules, intermediate filaments, basal bodies, and basal feet are in green, yellow, grey, and red respectively.
(related to Figure 1 and S1)
2. Reconstituted ultra-high voltage electron microscopic tomography images with extracted three dimensional models of the apical cytoskeletal networks and ciliary basal bodies in embryonic day 18 and postnatal days 2 and 7 mouse tracheal MCCs in the horizontal plane. Microtubules, intermediate filaments, basal bodies, and basal feet are in green, yellow, grey, and red, respectively.
(related to Figure 1)
3. Reconstituted ultra-high voltage electron microscopic tomography images with extracted three-dimensional models of the apical cytoskeletal networks and ciliary basal bodies in postnatal day 14 mouse tracheal MCCs in the horizontal plane. Microtubules, basal bodies, and basal feet are in green, grey, and red, respectively.
(related to Figure 2)
4. Reconstituted ultra-high voltage electron microscopic tomography images with extracted three-dimensional models of the apical cytoskeletal networks and ciliary basal bodies in Odf2 mutant adult (20 weeks old) mouse tracheal MCCs in the horizontal plane. Microtubules, intermediate filaments, basal bodies, and basal feet are in green, yellow, grey, and red, respectively.
(related to Figure 3)
5. Reconstituted ultra-high voltage electron microscopic tomography images with extracted three-dimensional models of the apical cytoskeletal networks in MDCK2 cells in the horizontal plane. Microtubules and intermediate filaments are in green and yellow, respectively.
(related to Figure 4)

Supplemental References

1. Konishi, S. *et al.* Directed Induction of Functional Multi-ciliated Cells in Proximal Airway Epithelial Spheroids from Human Pluripotent Stem Cells. *Stem Cell Reports* **6,** 18–25 (2016).
